# An Acetylene-Bridged 6,8-Purine Dimer as a Fluorescent Switch-On Probe for Parallel G-Quadruplexes**

**DOI:** 10.1002/anie.201207075

**Published:** 2012-12-13

**Authors:** Mehran Nikan, Marco Di Antonio, Keren Abecassis, Keith McLuckie, Shankar Balasubramanian

**Affiliations:** Department of Chemistry, The University of CambridgeLensfield Road, Cambridge, CB2 1EW (UK); Cambridge Research Institute, Cancer Research UK, Li Ka Shing CenterCambridge, CB2 0RE (UK); School of Clinical Medicine, The University of Cambridge, Addenbrooke's HospitalHills Road, Cambridge, CB2 0SP (UK)

**Keywords:** fluorescent probes, G-quadruplexes, purines, Sonogashira coupling

Exploiting chemistry to develop compounds capable of selective recognition of biomolecules or interfering with cellular processes is the essence of chemical biology.^1^ One example of such biologically relevant targets are nucleic acid G-quadruplexes.^2^ These non-canonical structures of DNA and RNA have been widely hypothesized to play a role in the regulation of crucial genomic functions, such as telomere maintenance,^3^ transcription,^4^ and translation.^5^ Recently, we showed that a small G-quadruplex-interacting molecule has the ability to activate the DNA damage response machinery in human cancer cells in a replication- and transcription-dependent manner.^6^

While much of the research in this field is focused on the development of G-quadruplex ligands for potential therapeutic use,^7^ it is also important to develop chemical probes that can specifically sense the formation of G-quadruplexes.^8^ Small molecules displaying fluorescence changes upon binding to G-quadruplexes have already been described.^9^ Nevertheless, reports on fluorescent scaffolds capable of discriminating between different G-quadruplex topologies are scarce, and those that discriminate generally fluoresce strongly with ds-DNA, thus hampering their further application.^10^

Herein, we describe the first example of a fluorescent switch-on probe termed acetylene-bridged purine dimer (APD), which is sensitive to the G-quadruplex structure and discriminates against ds-DNA. The APD molecule (**1**; see Scheme 2) structurally mimics half of a G-quartet with respect to its geometry and constituents and behaves as a selective fluorescent probe for parallel G-quadruplexes. The short acetylenic bond of APD retains the π conjugation required for fluorescence properties and maintains a spacing between two purines that is close to the size of a hydrogen bond in a G-quartet.

Our convergent approach for the preparation of **1** relies on the synthesis and coupling of fragments **6** and **7** depicted in Scheme 1 and Scheme 2. N9 alkylation of adenine by *N*-Boc-ethanolamine under Mitsunobu conditions and subsequent C8 bromination gave **2** and **3**, respectively.^11^ A facile diazotization–deamination of **3** under microwave irradiation by using THF as hydrogen-atom donor^12^ afforded **4**. Compound **4** was then subjected to Sonogashira coupling with triisopropylsilyl acetylene under microwave irradiation^13^ and deprotected to give **6**. The other fragment required for this synthesis, **7**, was prepared in a manner similar to that used for **4** using diiodomethane as the iodine source^14^ (Scheme 1). Our initial attempts for the cross-coupling of **6** and **7** by using common bases such as triethylamine or tetrabutylammonium fluoride (TBAF) led to the formation of known side products of cross-coupling^15^ or degradation of starting materials. Switching to caesium carbonate as the base effectively circumvented the problem and gave the Sonogashira product in 45 % yield after five hours at room temperature. In the final step, the *N*-Boc groups were cleaved under very mild conditions using tin tetrachloride^16^ to afford the desired product (Scheme 2).

**Scheme 1:**
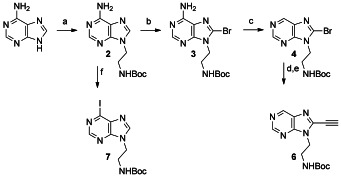
Synthetic route to **6** and **7**. Reagents and conditions: a) *N*-Boc ethanolamine, diisopropylazodicarboxylate (DIAD), PPh_3_, THF, 5 h, RT, 75 %; b) Br_2_, MeOH-1 m NaOAc, pH 4.3, 7 h, RT, 71 %; c) *n*-C_5_H_11_ONO, THF, MW (120 °C, 30 min), 62 %; d) triisopropylsilyl acetylene, [Pd(PPh_3_)_4_], CuI, Amberlite IRA-67, THF, MW (120 °C, 15 min), 89 %; e) TBAF-H_2_O (2:1), 15 min, RT, 85 %; f) *n*-C_5_H_11_ONO, CH_2_I_2_, MeCN, MW (120 °C, 15 min), 31 %. Boc=*tert*-butoxycarbonyl.

**Scheme 2:**
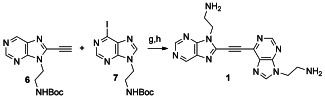
Sonogashira coupling followed by deprotection used to obtain APD (**1**). Reagents and conditions: g) [Pd(PPh_3_)_4_], CuI, Cs_2_CO_3_, THF, RT, 5 h, 45 %; h) SnCl_4_, EtOAc, 30 min, RT, 89 %.

We first investigated the binding potential of APD by using NMR spectroscopy on src1,^6^ a G-quadruplex formed from a G-rich sequence in the human *SRC* gene. The src1 sequence has a well-dispersed ^1^H NMR spectrum prior to the addition of APD, thus suggesting minimal polymorphism on the NMR time scale. After addition of the ligand, a significant upfield shift was observed in the imino region of the spectrum that is consistent with π–π stacking (Figure 1). Moreover, NMR titration revealed a 1:1 stoichiometry for ligand–quadruplex binding in solution. The inevitable repulsion between cationic side chains probably prevents two molecules of APD from sitting side-by-side for a 2:1 stoichiometry. Similar results were obtained for the titration of c-kit1 G-quadruplex (see the Supporting Information). Taken together, these results suggested that APD interacts with G-quadruplex DNA through end stacking.

**Figure 1:**
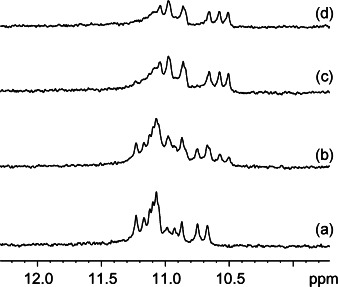
The imino region of the ^1^H NMR spectrum of the src1 sequence after the addition of a) 0 equiv, b) 0.5 equiv, c) 1 equiv, and d) 1.5 equiv of APD. Conditions: DNA (100 μm), KCl (100 mm), potassium phosphate buffer (20 mm; pH 7.4), RT, 500 MHz.

We then examined the fluorescence properties of APD. Interestingly, the molecule showed quite strong fluorescence emission in dichloromethane (*Φ*=0.19±0.02), whereas it showed negligible emission in water (*Φ*=0.003±0.001) at physiological pH and also in methanol (*Φ*=0.002±0.001) as summarized in Table 1. We hypothesized that such a marked reduction of the fluorescence emission in water or methanol could be due to a change in rotational diffusion about the acetylene bond.^17^ We found that APD emission properties were indeed very sensitive to increasing the solvent viscosity with poly(ethylene glycol) (PEG; Figure 2).

**Figure 2:**
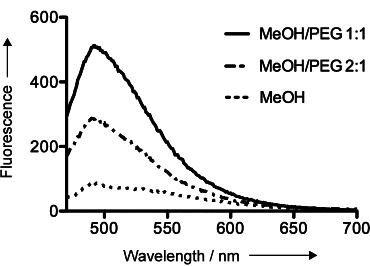
Fluorescence emission spectra recorded for: APD (5 μm) in MeOH, APD (5 μm) in a 2:1 MeOH/PEG solution, and APD (5 μm) in a 1:1 MeOH/PEG solution upon irradiation at 430 nm.

**Table 1:** Fluorescence quantum yields (*Φ*) and oligonucleotide concentration required to induce half-maximal fluorescence Δ*F*_50_

Sample[a]	*Φ*	Δ*F*_50_ [μm]
APD_Water_	0.003±0.002	–
APD 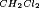	0.19±0.02	–
APD_MeOH_	0.002±0.001	–
APD_ds-DNA_	0.006±0.002	–
APD_H-Telo_	0.007±0.001	–
APD_TBA_	0.004±0.0001	–
APD_G3T3_	0.004±0.0001	2.6±0.1
APD_H-RAS_	0.007±0.003	2.2±0.3
APD_c-kit*_	0.003±0.002	–
APD_c-myc_	0.091±0.02	1.9±0.2
APD_c-kit1_	0.10±0.01	2.0±0.4
APD_BCL2_	0.12±0.02	1.1±0.2
APD_NRAS_	0.11±0.01	1.4±0.2
APD_TERRA_	0.12±0.01	0.5±0.2
APD_src1_	0.15±0.02	0.7±0.3

[a] The subscripts indicate either the solvent in which APD was measured (first three entries), or the oligonucleotide that was added to APD in phosphate buffer (for oligonucleotide sequences see the Supporting Information).

We then investigated whether the addition of G-quadruplexes could similarly lock APD in a fluorescent active conformation. To address this question, we first examined the UV/Vis absorption spectra of the APD in the presence and absence of different G-quadruplex structures. While the spectrum of APD was not noticeably affected in the presence of h-Telo, which is a mixed-type DNA G-quadruplex structure,^18^ the addition of c-kit1,^19^ which is a parallel DNA G-quadruplex structure,^20^ induced a very significant change (Figure 3). The spectral changes stopped after the addition of one equivalent of c-kit1, which is consistent with the 1:1 stoichiometry measured with NMR spectroscopy.^21^

**Figure 3:**
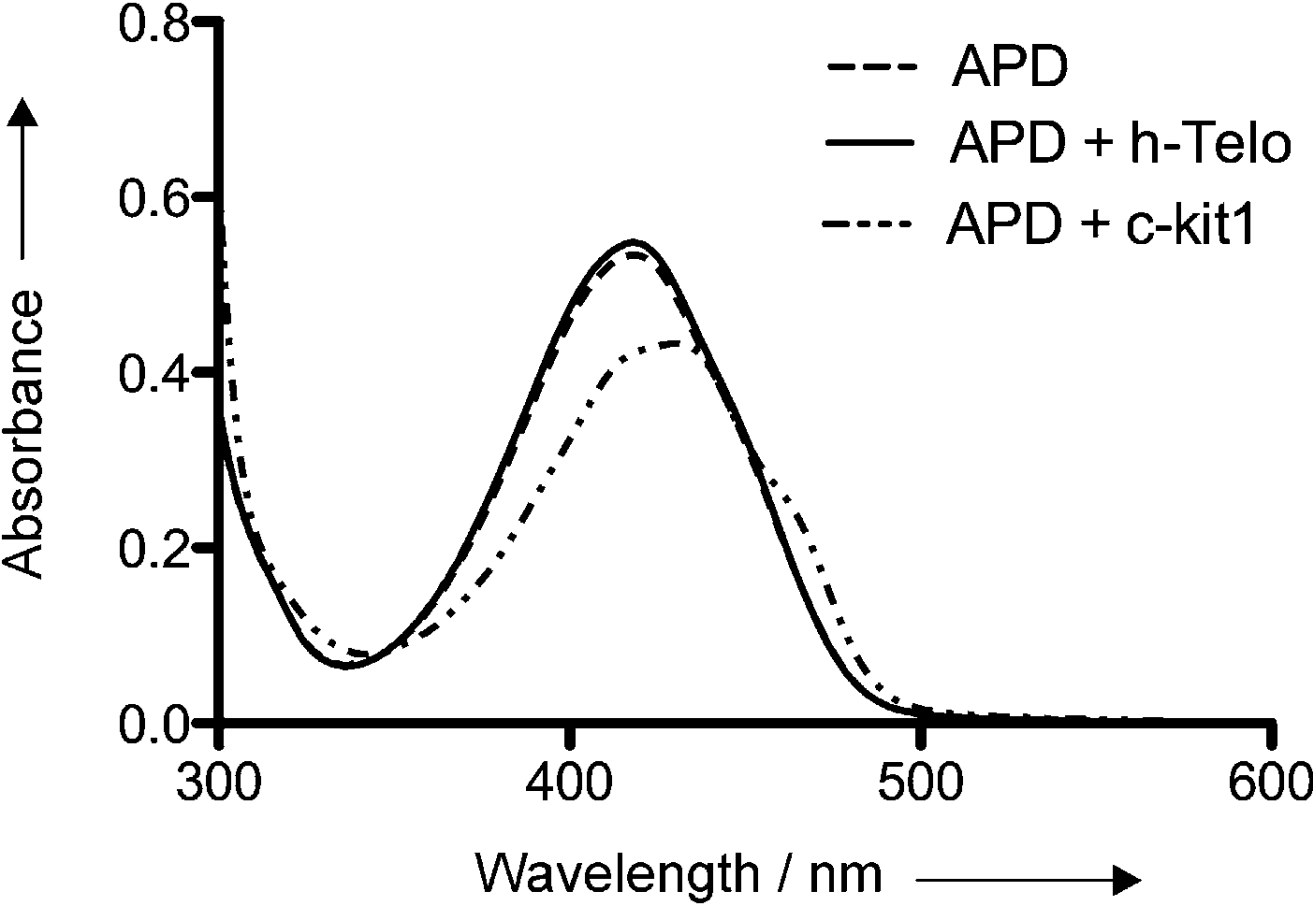
UV spectra of APD, of APD after addition of pre-annealed h-Telo (1 equiv), and of APD after addition of pre-annealed c-kit1 (1 equiv). Conditions: APD (25 μm), potassium phosphate buffer (50 mm; pH 7.4), KCl (50 mm), RT.

The observed decrease in the UV absorption (*ε*_APD_=19 386 L mol^−1^ cm^−1^; *ε*_APD-ckit1_=14 167 L mol^−1^ cm^−1^ at 430 nm) along with red shift (Δ*λ*≍8 nm) provided the first evidence that the photophysical properties of APD are changed upon interaction with c-kit1. The UV spectrum recorded under these conditions closely resembles the one obtained for APD in a 1:1 MeOH/PEG mixture (see the Supporting Information); thus suggesting that APD could act as a fluorescent switch-on probe for parallel G-quadruplexes. To ascertain the generality of this finding, we carried out fluorescence titrations of APD with several G-quadruplexes, as well as ds-DNA (Figure 4). A significant fluorescence enhancement was observed when APD was treated with the DNA G-quadruplexes c-kit1,^19^ c-myc,^22^ and src1^6^ and the RNA G-quadruplexes BCL2,^23^ NRAS,^24^ and TERRA (telomeric tandem repeat containg RNA)^25^ that have all been shown to form a parallel G-quadruplex (see the Supporting Information for the diagnostic CD spectra). In contrast, we observed negligible fluorescence enhancement for h-Telo, a mixed-type DNA G-quadruplex structure,^18^ and antiparallel DNA G-quadruplexes: H-RAS,^26^ c-kit*,^27^ G_3_T_3_,10a and TBA (thrombin binding aptamer),^28^ under the experimental conditions. Notably, no fluorescence enhancement was observed when titrating APD with ds-DNA. To further confirm this observation, we carried out a control experiment in which a preformed complex of TERRA/APD was treated with 20-fold excess of h-Telo. We detected negligible changes in the fluorescence emission, thus indicating a large difference in APD binding to h-Telo and TERRA (see the Supporting Information).

**Figure 4:**
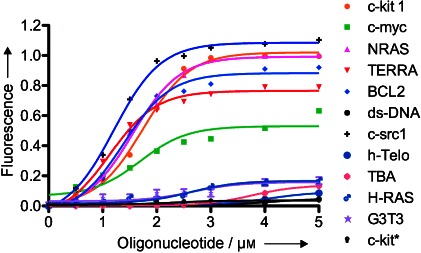
Fluorescence titration of APD with preannealed DNA/RNA G-quadruplexes and ds-DNA. Conditions: APD (500 nm), KCl (50 mm), potassium phosphate buffer (50 mm; pH 7.4), RT. The solutions were excited at 430 nm and the fluorescence intensity recorded at 510 nm.

The concentrations of oligonucleotides required to give half-maximal fluorescence with APD along with quantum yield values are summarized in Table 1. The quantum yields reported here clearly show that fluorescence enhancement is always more pronounced for parallel G-quadruplexes. Notably, the fluorescence emission of G-quadruplex-bound APD was decreased upon addition of a competitive G-quadruplex-binding ligand (PDS)^6^ in a dose-dependent manner (see the Supporting Information).

As an example of application, we set to demonstrate the potential of APD as a G-quadruplex staining reagent in electrophoresis gels. Fluorescent dyes such as ethidium bromide have been used routinely to stain DNA and RNA. There has also been some success in visualizing G-quadruplexes against ds-DNA.9c,f,g,i We sought to take this approach further by achieving topology-specific targeted staining of G-quadruplexes. We employed TERRA, h-Telo, c-kit1, and ds-DNA as our test system at concentrations of 2.5 μm. Aliquots of a stock solution of APD were then added to each sample to a final concentration of 500 nm. As shown in Figure 5 a, we were only able to detect bands corresponding to TERRA and c-kit1 that form parallel G-quadruplexes, whereas we found no staining for both h-Telo and ds-DNA. A dose-response experiment showed that TERRA G-quadruplex is still detectable at 0.25 μm concentration (Figure 5 b).

**Figure 5:**
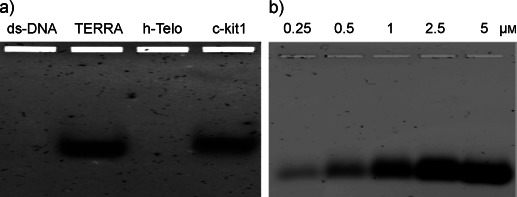
a) Electrophoresis staining of ds-DNA, TERRA, h-Telo, and c-kit1 with APD. b) Dose-response staining of TERRA G-quadruplex with APD. Conditions: 2.5 % agarose gel, 1X Tris–acetate–EDTA (TAE) buffer containing KCl (20 mm), oligo (2.5 μm), APD (500 nm), total volume 20 μL, 4 °C.

The strong discrimination observed in the fluorescence emission of APD upon treatment with TERRA (telomeric-repeat containing RNA) and h-Telo (telomeric-repeat containing DNA) G-quadruplexes is of particular importance in the light of recent findings suggesting that these two structures may exist concurrently and be involved in regulation of different processes at telomeres.^29^ This result further supports our previous observations and further highlights the potential of APD as a topology-specific diagnostic probe for G-quadruplexes.

In conclusion, we described the synthesis and photophysical properties of an acetylene-bridged 6,8-purine dimer, termed APD, and showed that the fluorescence emission of this molecule increases significantly upon binding to parallel G-quadruplexes. Fluorescence titrations suggested that APD binds to several parallel G-quadruplexes and NMR spectroscopy experiments revealed that end stacking is the preferred mode of binding. The selective fluorescence of APD can be rationalized in terms of the formation of a fluorescent complex with parallel G-quadruplexes in comparison with antiparallel G-quadruplexes. The photophysical characterization of APD revealed that the modulation of rotational diffusion properties plays a role in this regard. The exact structure of these complexes, however, remains to be further revealed. Owing to its unique features APD represents a potentially valuable tool to selectively stain parallel G-quadruplexes.

## Supplementary material

As a service to our authors and readers, this journal provides supporting information supplied by the authors. Such materials are peer reviewed and may be re-organized for online delivery, but are not copy-edited or typeset. Technical support issues arising from supporting information (other than missing files) should be addressed to the authors.
