# Supplementary material for: An Acetylene-Bridged 6,8-Purine Dimer as a Fluorescent Switch-On Probe for Parallel G-Quadruplexes
Source: Angew Chem Int Ed Engl. 2012 Dec 13;52(5):1428–31. doi: 10.1002/anie.201207075 (PMC3652027; doi:10.1002/anie.201207075)
Supplement: Supplementary file 1 [file anie0052-1428-SD1.pdf]

Angewandte Chemie
Eine Zeitschrift der Gesellschaft Deutscher Chemiker

Supporting Information
© Wiley-VCH 2013
69451 Weinheim, Germany

An Acetylene-Bridged 6,8-Purine Dimer as a Fluorescent Switch-On Probe for Parallel G-Quadruplexes**

*Mehran Nikan, Marco Di Antonio, Keren Abecassis, Keith McLuckie, and Shankar Balasubramanian**

anie_201207075_sm_miscellaneous_information.pdf

Experimental

General

All reactions were conducted under an argon atmosphere using anhydrous freshly distilled solvents unless otherwise stated. THF and acetonitrile were dried and purified by distillation from sodium/benzophenone and CaH_2 respectively. Flash chromatography was performed using 230-400 mesh silica gel from Sigma-Aldrich. Analytical thin-layer chromatography (TLC) was performed using silica gel 60 F_{254} using UV light as visualizing agent. Preparative thin layer chromatography (PLC) was carried out on similar plates (1 mm) from Merck. ^1H and ^{13}C NMR spectra were recorded on Bruker DRX-400, DPX-400, DRX-500 instruments using residual solvent as reference. High-resolution mass spectra were obtained on a Waters Micromass® Q-Tof (ESI) spectrometer. HPLC purification was performed on a Varian ProStar instrument equipped with a Pursuit C18, 5*μ* column (250 × 21.2 mm) using a linear elution gradient from 90% H_2O (containing 0.1% TFA) to 100% MeCN in 30 min at a flow rate of 12.0 ml/min. Microwave reactions were conducted using a single-mode CEM discover microwave unit. The maximum microwave power was set at 140 W and the pressure limit at 16 bar. All reactions were performed in crimp sealed thick walled glass vessels (capacity 10 mL or 30 mL). The reaction mixture was stirred using a Teflon-coated magnetic stirring bar and the temperature was monitored using an infrared sensor.

Compounds **2**, **3** were synthesized according to a reported procedure. [1]

Synthesis of purine **4**

To a suspension of **3** (100 mg, 0.28 mmol) in dry degassed THF (3 mL) in a 10 mL sealed Microwave tube was added 0.260 mL of *n*-amyl nitrite (1.90 mmol) *via* a microsyringe. The reaction mixture was stirred under microwave irradiation at 120 °C for 30 min. After reaching completion, the reaction mixture was cooled to room temperature and the solvent was removed *in vacuo*. The product was then purified by column chromatography on silica gel using EtOAc as the eluent, to afford **4** as a white solid (yield 65%).

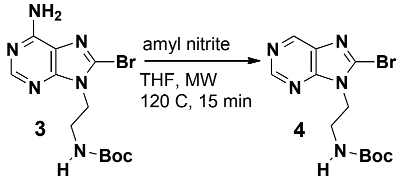

^1H NMR (500 MHz, $CDCl_3$, 298 K) δ_H 9.01 (s, 1 H), 8.92 (s, 1 H), 4.84 (s, broad, NH), 4.46 (t, 2 H), 3.62 (t, 2H), 1.32 (s, 9 H) ppm; ^{13}C NMR (500 MHz, $CDCl_3$, 298 K) δ_c 155.8, 152.8, 152.7, 147.2, 134.7, 134.5, 80.0, 44.5, 39.7, 28.3 ppm; HRMS (ES) calculated: 341.0596 for $C_{12}H_{16}BrN_5O_2$; found: [M+H$^+$] 342.0549.

Synthesis of purine **5**

4 (171 mg, 0.50 mmol), $Pd(PPh_3)_4$ (5.5 mg, 0.005 mmol, 1 mol%), CuI (2.2 mg, 0.011 mmol, 2 mol%), and Amberlite IRA-67 (1.20 g, 6.25 mmol) were suspended in dry degassed THF (3 mL) in a 10 mL sealed Microwave tube under argon. To this solution was added 0.300 mL of triisopropylsilyl acetylene (1.30 mmol) *via* a microsyringe and the mixture was stirred under microwave irradiation at 120 °C for 15 min. After reaching completion, the reaction mixture was cooled to room temperature and the solvent was removed *in vacuo*. The product was then purified by column chromatography on silica gel using Hex-EtOAc (1:1) as the eluent, to afford **5** as a pale yellow oily solid (yield 89%).

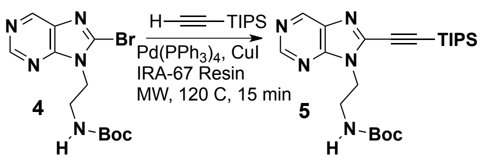

^{1}H NMR (500 MHz, $CDCl_3$, 298 K) δ_H 9.06 (s, 1 H), 8.96 (s, 1 H), 4.90 (s, broad, NH), 4.50 (t, 2 H), 3.64 (t, 2H), 1.30 (s, 9 H), 1.24-1.15 (m, 21 H) ppm; ^{13}C NMR (500 MHz, $CDCl_3$, 298 K) δ_c 155.6, 153.5, 151.6, 148.7, 139.3, 133.6, 103.1, 94.2, 79.8, 44.0, 40.2, 28.3, 18.7, 11.2 ppm; HRMS (ES) calculated: 443.2717 for $C_{23}H_{37}N_5O_2Si$; found: [M+H$^+$] 444.2837.

Synthesis of purine **6**

5 (60 mg, 0.135 mmol) was dissolved in TBAF (1M solution in THF)-H_2O (3 mL, 2:1) and stirred at room temperature for 15 min. Solvent was removed *in vacuo*. The residue was dissolved in 5 mL of ethyl acetate, washed three times with 10 mL of water, and dried with anhydrous $MgSO_4$. After removal of the solvent *in vacuo*, the product was purified by column chromatography on silica gel using EtOAc as the eluent, to afford **6** as a white solid (yield 81%)

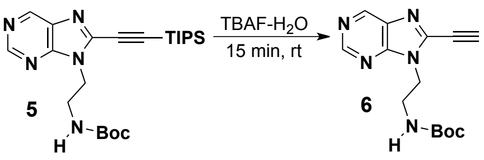

^{1}H NMR (500 MHz, $CDCl_3$, 298 K) δ_H 9.08 (s, 1 H), 8.98 (s, 1 H), 4.96 (s, broad, NH), 4.54 (t, 2 H), 3.64 (t, 2 H), 3.60 (s, 1 H), 1.35 (s, 9 H) ppm; ^{13}C NMR (500 MHz, $CDCl_3$, 298 K) δ_c 155.8, 153.6, 151.3, 148.9, 138.8, 133.5, 85.6, 79.9, 72.3, 43.9, 40.0, 28.4 ppm; HRMS (ES) calculated: 287.1382 for $C_{14}H_{17}N_5O_2$; found: [M+H$^+$] 288.1485.

Synthesis of purine **7**

To a suspension of **2** (117 mg, 0.42 mmol) in dry degassed MeCN (3 mL) in a 10 mL sealed Microwave tube was added 1 mL of diiodomethane (12.4 mmol) and 0.260 mL of *n*-amyl nitrite (1.90 mmol) under argon using a microsyringe. The reaction mixture was stirred under microwave irradiation at 120 °C for 15 min. After reaching completion, the reaction mixture was cooled to room temperature and the solvent was removed *in vacuo*. The residue was dissolved in EtOAc and washed with saturated $Na_2S_2O_3$ and water. The organic layer was evaporated *in vacuo* and the crude mixture was purified by column chromatography on silica gel using EtOAc as the eluent, to afford **7** as a white solid (yield 31%).

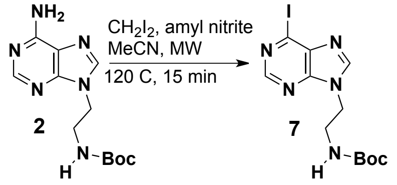

^{1}H NMR (500 MHz, $CDCl_3$, 298 K) δ_H 8.62 (s, 1 H), 8.11 (s, 1 H), 4.83 (s, broad, NH), 4.44 (t, 2 H), 3.58 (t, 2

H), 1.38 (s, 9 H) ppm; ^{13}C NMR (500 MHz, $CDCl_3$, 298 K) δ_c 156.1, 152.3, 148.4, 145.3, 138.9, 122.4, 77.6, 44.5, 40.5, 28.6 ppm; HRMS (ES) calculated: 389.0349 for $C_{12}H_{16}IN_5O_2$; found: [$M+H^+$] 390.0444.

<u>Synthesis of APD **1**</u>

6 (12 mg, 0.041 mmol), **7** (14 mg, 0.036 mmol), $Pd(PPh_3)_4$ (5.5 mg, 0.005 mmol, 14 mol%), CuI (2.0 mg, 0.010 mmol, 28 mol%), and Cs_2CO_3 (40 mg, 0.123 mmol) were suspended in dry degassed THF (3 mL) in a 5 mL RB flask under argon. The reaction mixture was stirred at rt for 5 h. The solvent was removed *in vacuo* and the crude mixture was purified on preparative TLC plates using EtOAc-MeOH (3:1) as the eluent, to afford the product in the *N*-Boc protected form.
<u>Deprotection</u>: 20 μL of tin tetrachloride (0.116 mmol) was added to a suspension of 7 mg of *N*-Boc protected product (0.012 mmol) in dry EtOAc (0.5 mL). The resulting clear solution was stirred at rt for 30 min until TLC (EtOAc-MeOH 3:1) showed complete consumption of the starting material. The product was precipitated by the addition of diethylether (3 mL), collected by filtration and purified by HPLC (see general experimental for conditions) to afford **1** as yellow TFA salt (yield 40% over two steps).

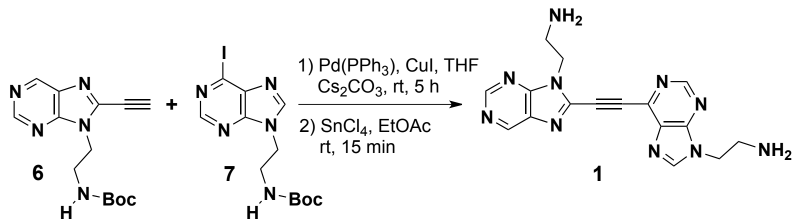

^{1}H NMR (500 MHz, D_2O, 298 K) δ_H 8.67 (s, 1 H), 8.41 (s, 1 H), 8.37 (s, 1 H), 8.35 (s, 1 H), 6.27 (H-bonded NH), 4.61 (t, 2 H), 4.35 (t, 2 H), 3.91 (t, 2 H), 3.60 (t, 2 H) ppm; ^{13}C NMR (500 MHz, D_2O, 298 K) δ_c 151.2, 150.6, 149.4, 149.1, 148.0, 147.7, 145.9, 143.2, 132.7, 125.7, 94.4, 86.2, 41.8, 40.0, 39.0, 38.6 ppm; HRMS (ES) calculated: 348.1559 for $C_{16}H_{16}N_{10}$; found: [$M+H^+$] 349.1654.

[1] S. Vancalenbergh, C. L. M. J. Verlinde, J. Soenens, A. Debruyn, M. Callens, N. M. Blaton, O. M. Peeters, J. Rozenski, W. G. J. Hol, P. Herdewijn, *J. Med. Chem.* **1995**, *38*, 3838-3849.

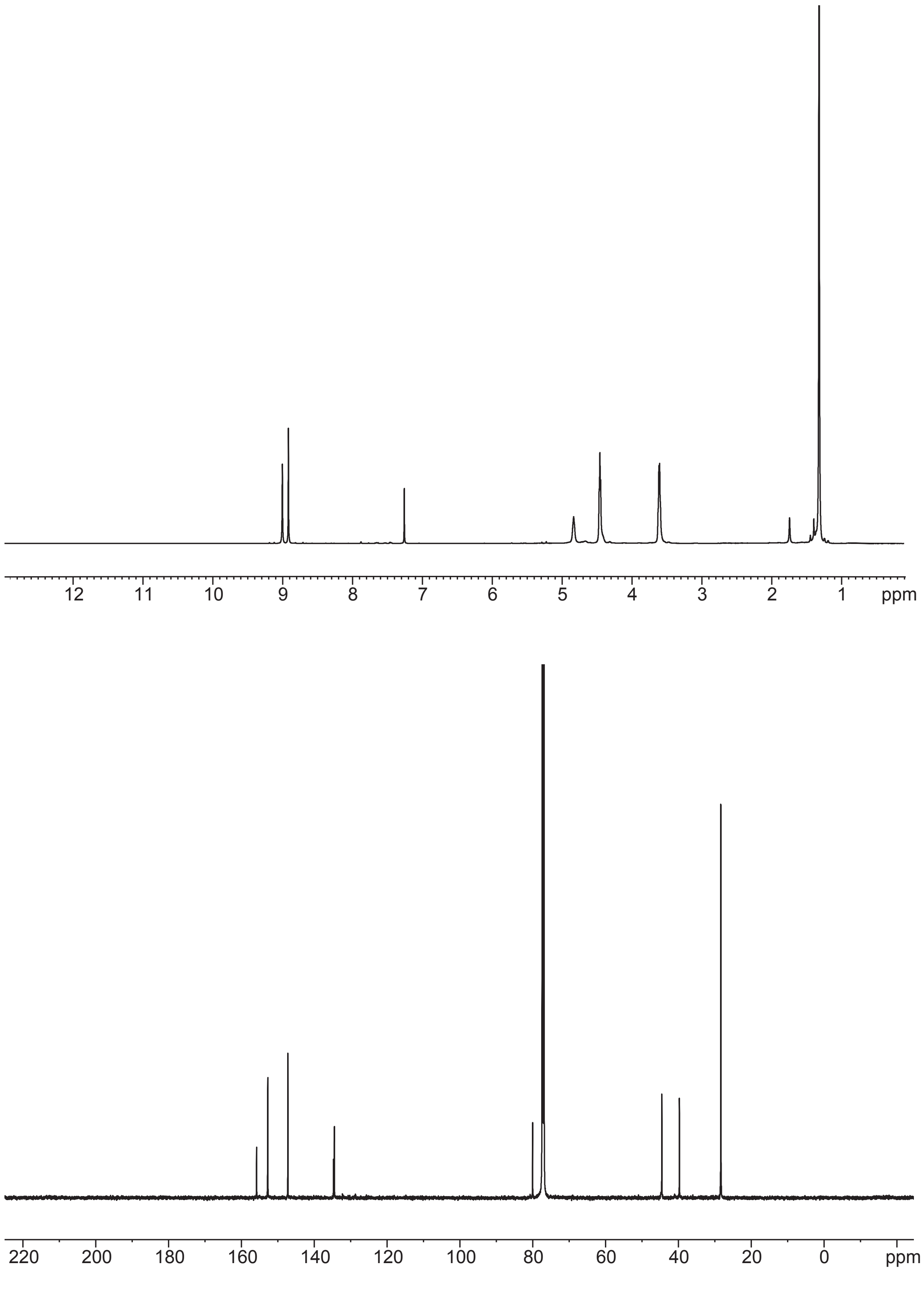

Figure S1: ^{1}H and ^{13}C spectra of **4** at 500 MHz at 298 K in $CDCl_3$.

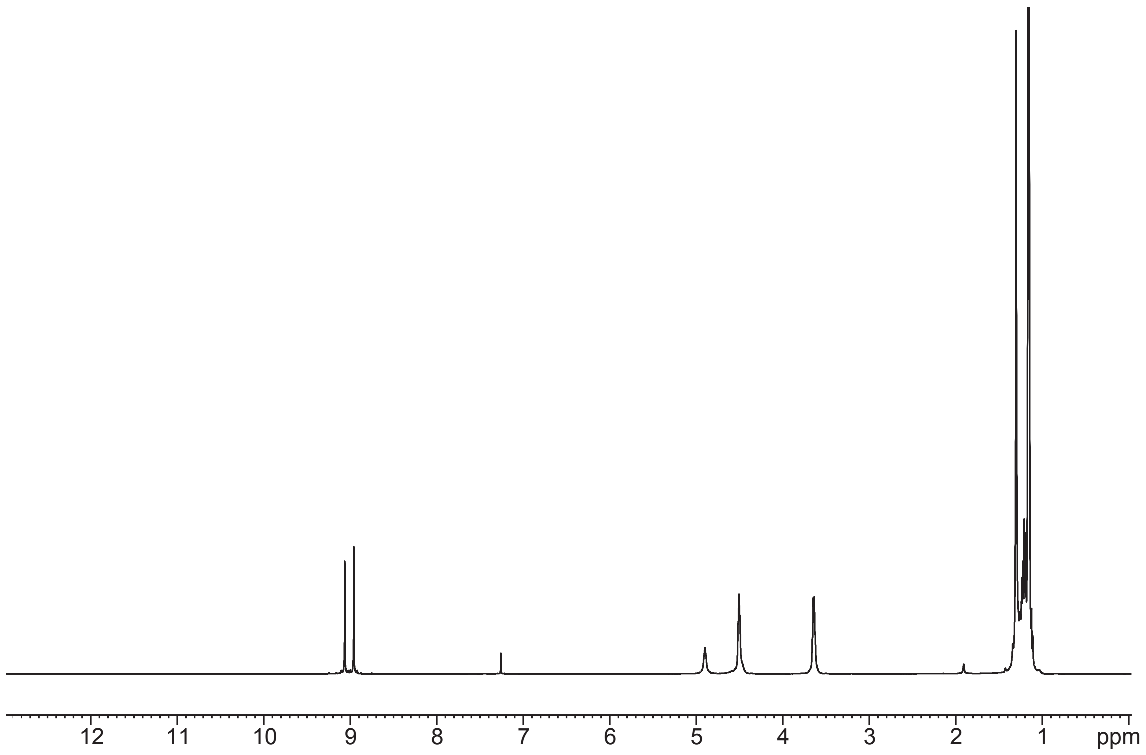

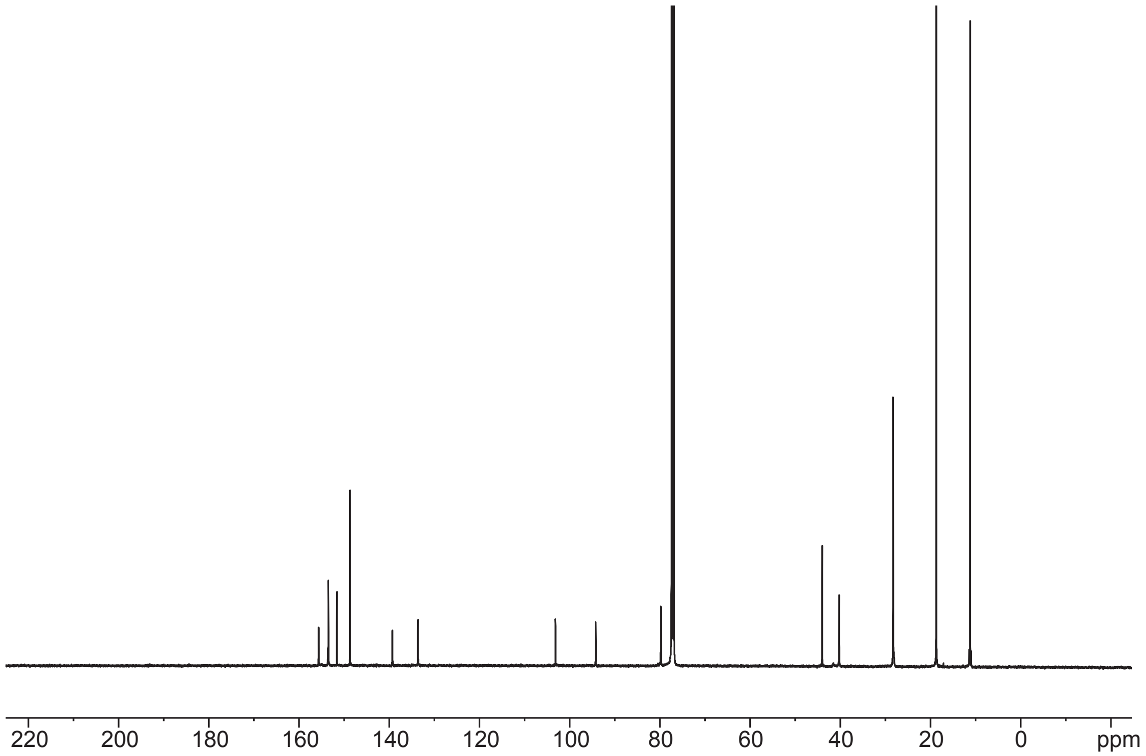

Figure S2: ^{1}H and ^{13}C spectra of **5** at 500 MHz at 298 K in $CDCl_3$.

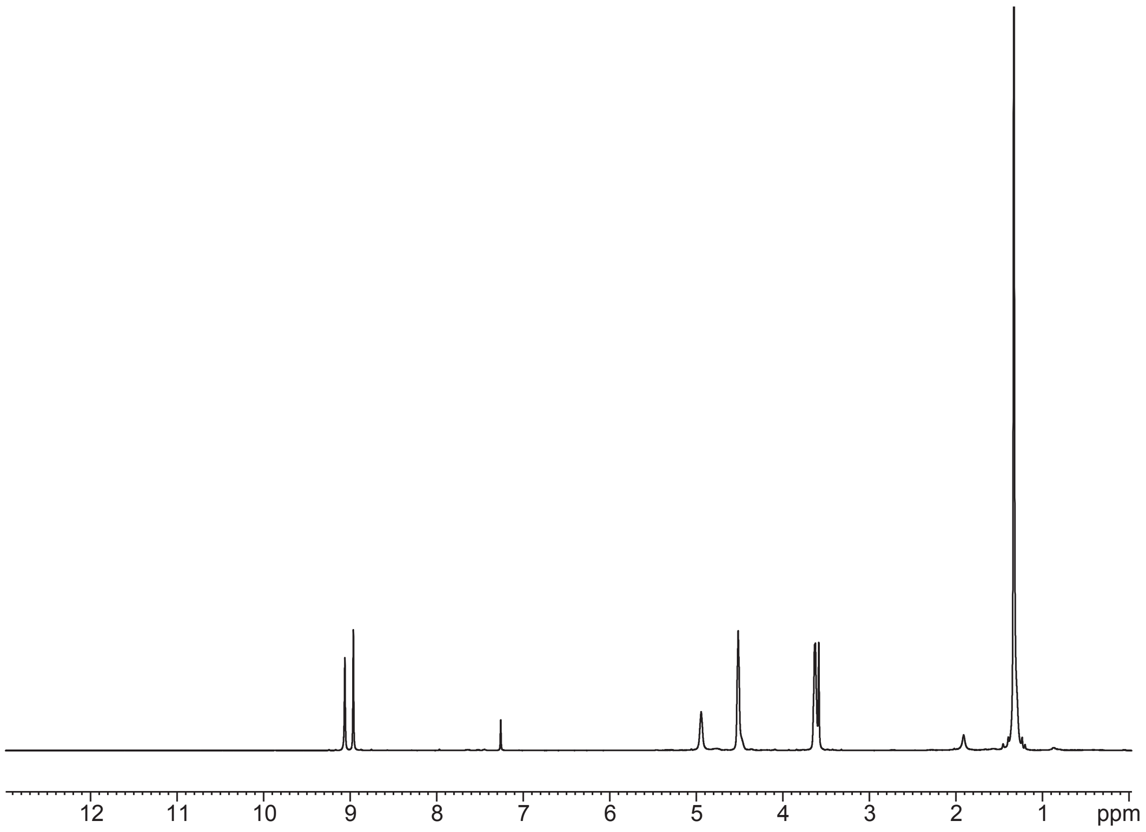

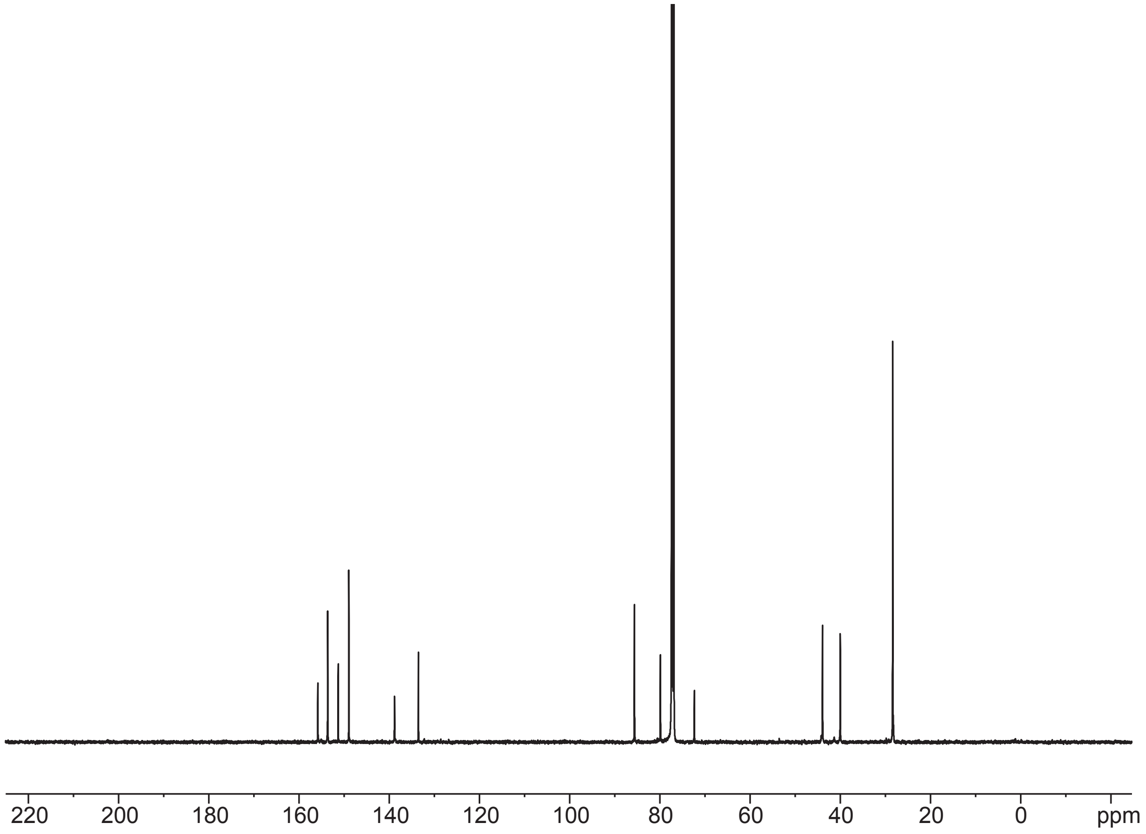

Figure S3: ^{1}H and ^{13}C spectra of **6** at 500 MHz at 298 K in $CDCl_3$.

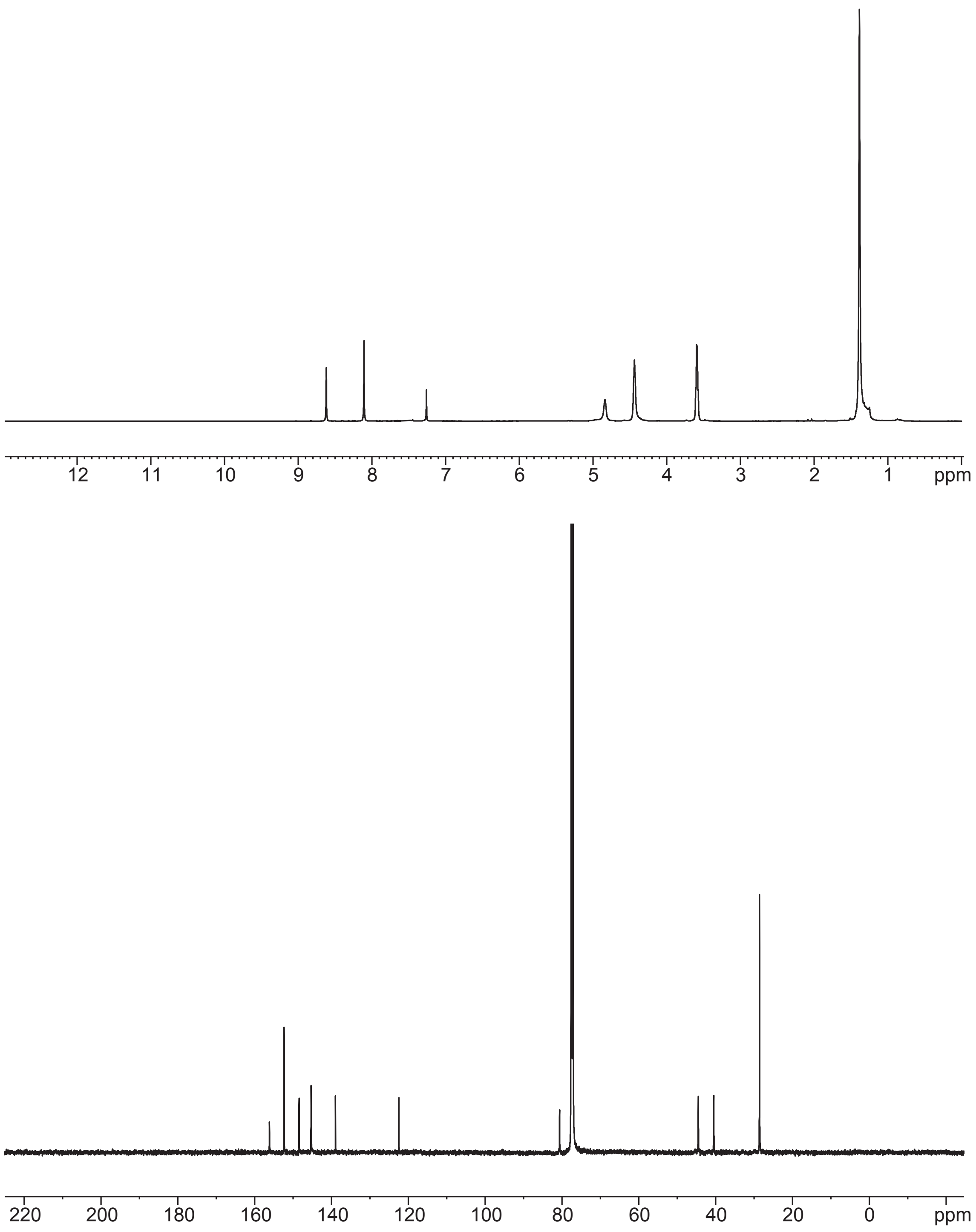

Figure S4: ^{1}H and ^{13}C spectra of **7** at 500 MHz at 298 K in $CDCl_3$.

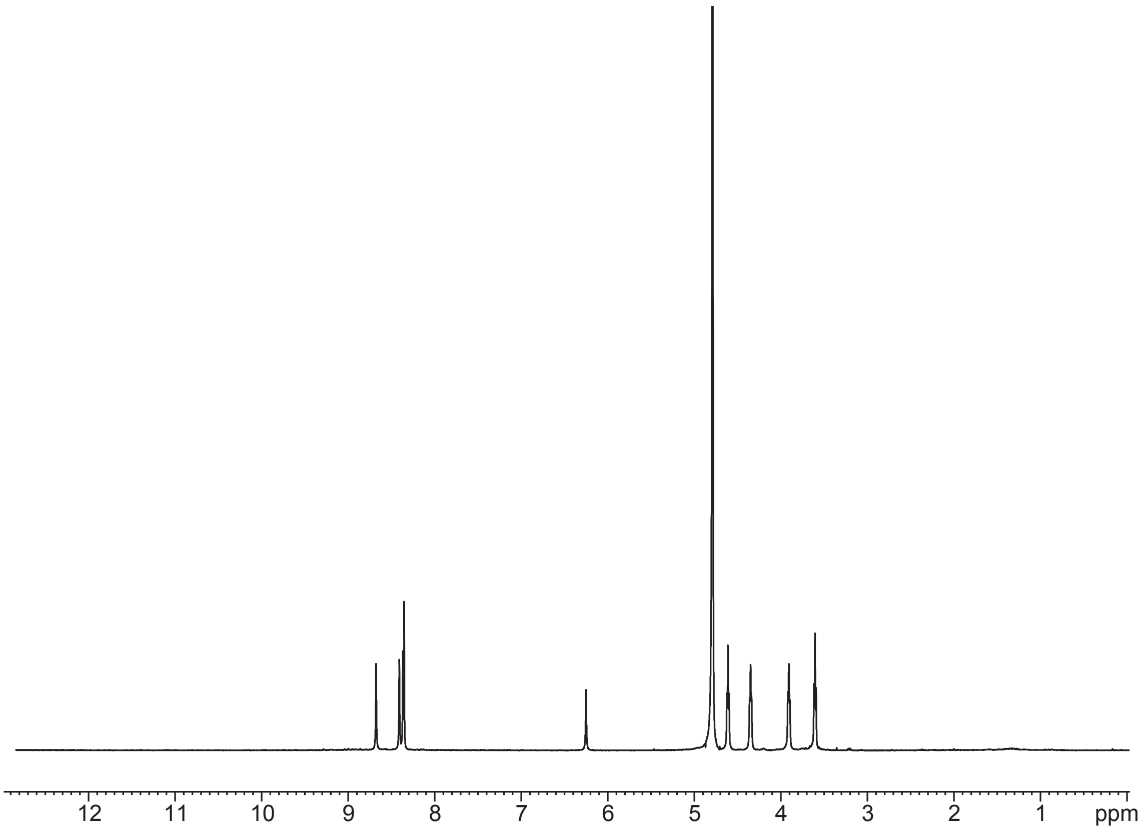

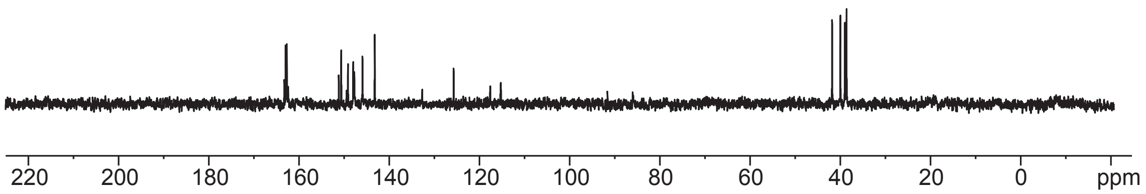

Figure S5: ^{1}H and ^{13}C spectra of APD (**1**) at 500 MHz at 298 K in D_2O.

Oligonucleotides

Oligonucleotides were purchased from Eurogentec® Ltd. as HPLC purified samples. The following sequences were used for the experiments: h-Telo (DNA) 5′-A-GGG-TTA-GGG-TTA-GGG-TTA-GGG-T-3′; c-myc (DNA) 5′-T-TGA-GGG TGG GTA GGG TGG-GTA-AA-3′; TERRA (RNA) 5′-A-GGG-UUA-GGG-UUA-GGG-U-3′; c-kit1 (DNA) 5′-AGG-GAG-GGC-GCT-GGG-AGG-AGG-G-3′; src1 (DNA) 5′-GGG-CGG-CGG-GCT-GGG-CGG-GG-3′; TBA (DNA) 5′-GGT-TGG-TGT-GGT-TGG-3′; NRAS (RNA) 5′-GGG-AGG-GGC-GGG-UCU-GGG-3′; BCL2 (RNA) 5′-GGG-GGC-CGU-GGG-GUG-GGA-GCU-GGG-G-3′; G_3T_3 (DNA) 5′-GGG-TTT-GGG-TTT-GGG-TTT-GGG-3′; H-RAS (DNA) 5′-TC-GGG-TTG-CGG-GCG-CAG-GGC-ACG-GGC-G-3′; c-kit* (DNA) 5′-GGC-GAG-GAG-GGG-CGT-GGC-CGG-C-3′; ds-DNA self-complementary sequence 5′-CAA-TCG-GAT-CGA-ATT-CGA-TCC-GAT-TG-3′. Oligonucleotides were annealed in their respective buffers prior to each measurement by heating at 95 °C for 10 min and then were allowed to cool down to 4 °C overnight.

UV-visible spectroscopy

UV-Vis spectra were recorded on a Varian Cary 100-Bio at 298 K using a 10 mm path length quartz cuvette. Titrations were performed by adding a solution of pre-annealed oligonucleotide (1 mM stock solution) to a solution of APD (25 μM) in PBS buffer (50 mM, pH 7.4) containing 50 mM KCl. Samples were left to equilibrate after each addition to ensure a stable readout.

Fluorescence spectroscopy and quantum yield calculations

Fluorescence measurements were performed using a Varian Cary Eclipse Spectro-Fluorometer. Emission spectra were recorded using a 10 mm path length quartz cuvette by exciting the samples at 430 nm and recording the emissions over the spectral range of 480-600 nm. (slit emission and excitation were set at 10 nm). Fluorescence titrations were performed by adding solutions of pre-annealed oligonucleotides (100 μM and 1 mM) to a solution of APD (0.5 μM) in PBS buffer (50 mM, pH 7.4) containing 50 mM KCl. Samples were left to equilibrate after each addition to ensure a stable readout. All titrations were performed in triplicates.

Quantum yields were calculated using fluorescein as standard (ϕ = 0.95). Emission spectra of APD and fluorescein were recorded (25 μM) in a 50 mM potassium phosphate buffer (pH 7.4) containing 50 mM KCl using a λ_{ext} = 430nm. Oligonucleotides pre-anelled in this buffer were added to the APD solution to the final concentration of 100 μM. ODs of these samples were recorded at 430 nm and the quantum yield values were calculated according to the following equation:

$$\phi_{sample} = \phi_{ref}\,(\mathbf{A_{sample}/A_{Ref}})*(\mathbf{OD_{Ref}/OD_{Sample}})$$

Where: ϕ_{ref} is the quantum yield of the reference; $\mathbf{A_{sample}}$ and $\mathbf{A_{ref}}$ are the area underneath the emission spectra of the sample and the reference respectively and $\mathbf{OD_{ref}}$ and $\mathbf{OD_{sample}}$ are the optical density of the reference and the sample respectively measured at the excitation wavelength.
All the experiments have been performed in triplicates.

Circular dichroism spectroscopy

CD spectra were recorded on an Applied Photo-physics Chirascan circular dichroism spectropolarimeter using a 1 mm path length quartz cuvette. CD measurements were performed at 298 K over a range of 200-340 nm using a response time of 1 s, 1 nm pitch and 0.5 nm bandwidth. The recorded spectra represent a smoothed average of three scans, zero-corrected at 320 nm and normalized (Molar ellipticity θ is quoted in

10^5 deg cm^2 $dmol^{-1}$). The absorbance of the buffer was subtracted from the recorded spectra. Oligonucleotides were dissolved in lithium cacodilate buffer (100 mM, pH 7.2) containing 100 mM of KCl and 1 mM EDTA to the concentration of 10 µM. The oligonucleotides were annealed following the above-mentioned conditions prior to measurements.

FRET-melting assays

100 µM stock solutions of oligonucleotides were prepared in molecular biology grade DNase-free water. Further dilutions were carried out in 60 mM potassium cacodylate buffer, pH 7.4. FRET experiments were carried out with a 200 nM oligonucleotide concentration. All labeled DNA oligonucleotides were supplied by IBA® GmbH. Dual fluorescently labeled DNA oligonucleotides used in these experiments: H-Telo (5′-FAM-GGG TTA GGG TTA GGG TTA GGG-TAMRA-3′), c-kit1 (5′-FAM-GGG AGG GCG CTG GGA GGA GGG-TAMRA-3′), c-kit2 (5′-FAM-GGG CGG GCG CGA GGG AGG GG-TAMRA-3′), c-myc (5′-FAM-TGA GGG TGG GTA GGG TGG GTA A-TAMRA-3′), KRAS (5′-FAM AGG GCG GTG TGG GAA GAG GGA AGA GGG GGA GG-TAMRA-3′, TERRA (5′-FAM-GGG UUA GGG UUA GGG UUA GGG-TAMRA-3′) and ds-DNA (5′-FAM-TAT AGC TAT A-HEG-T ATA GCT ATA-TAMRA-3′) which is a dual-labeled 20-*mer* oligonucleotide comprising a self-complementary sequence with a central polyethylene glycol linker able to fold into a hairpin. The donor fluorophore was 6-carboxyfluorescein (FAM) and the acceptor fluorophore was 6-carboxytetramethylrhodamine (TAMRA). The dual-labeled oligonucleotides were annealed at a concentration of 400 nM by heating at 94 °C for 10 min followed by slow cooling to rt at a controlled rate of 0.1 °C/min. 96-well plates were prepared by addition of 50 µl of the annealed DNA solution to each well, followed by 50 µl solution of APD at the appropriate concentration. Measurements were made in duplicate with an excitation wavelength of 483 nm and a detection wavelength of 533 nm. Final analysis of the data was carried out using Prism 5 data analysis and graphing software (Prism®).

Nuclear magnetic resonance spectroscopy

Titration with **1** was performed on a 500 MHz TCI-ATM Cryo instrument at 298 K and the spectra were recorded immediately after each addition. Water suppression was achieved using excitation sculpting. Oligonucleotide samples were dissolved in phosphate buffer (20 mM, pH 7.4) containing 100 mM KCl to the concentrations of 100-200 *µ*M (1:9 mixture of D_2O/H_2O), and were annealed following the above-mentioned conditions prior to data acquisition.

ITC Measurements

ITC titration was performed with a NanoCal TA instrument equipped with a 250 µL syringe and 950 µL cell, using a stirring speed of 200 rpm and a temperature of 298 K. Titrations were performed by adding APD (1 mM) to a solution of pre-annealed c-kit1 (0.1 mM) in a 50 mM PBS (pH 7.4) containing 50 mM KCl. The oligonucleotide was titrated by adding APD in 20 successive injections of 5 µL each.

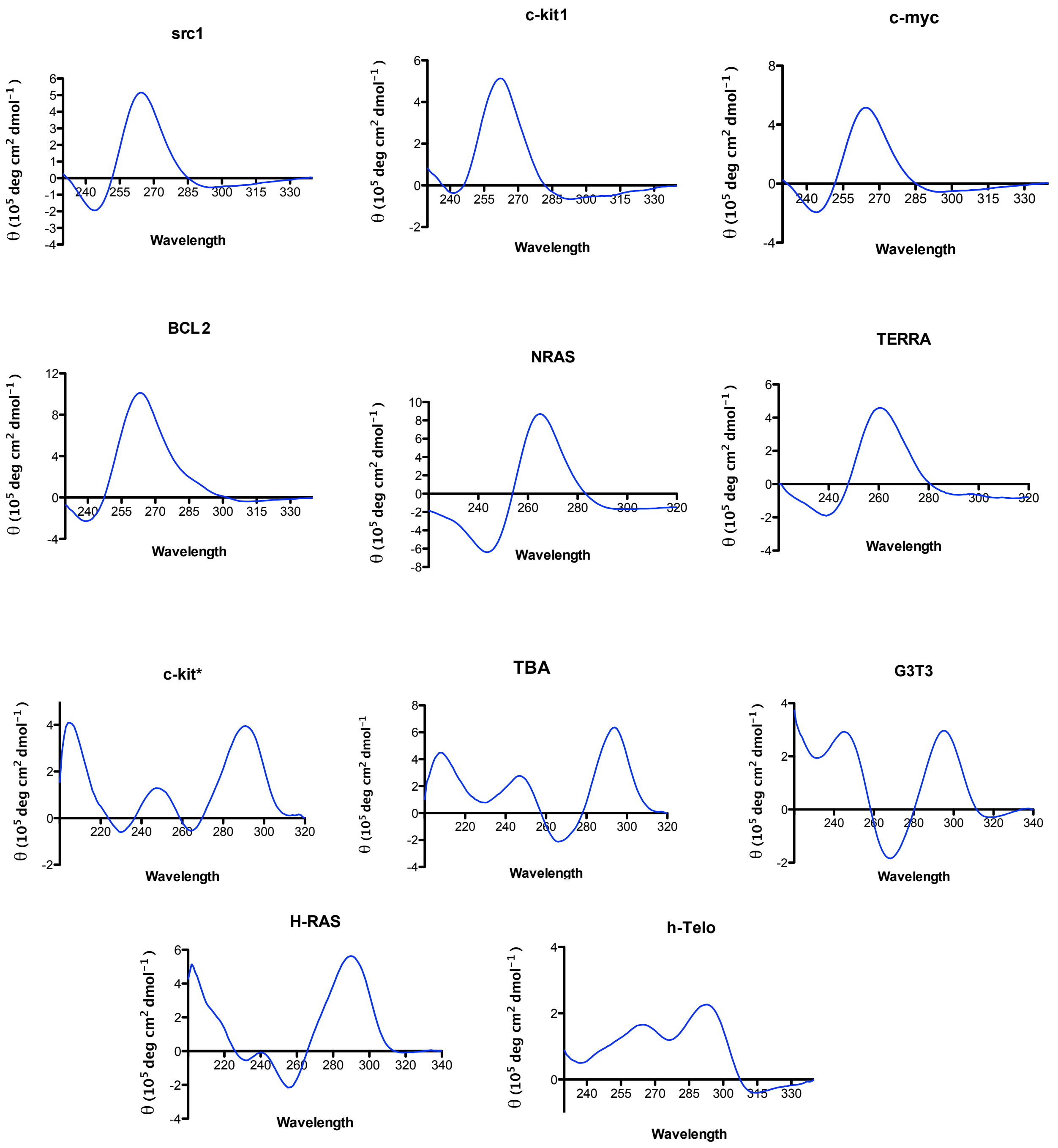

Figure S6: CD signatures of a 10 μM solution of G-quadruplex-forming oligonucleotides in lithium cacodilate buffer (100 mM, pH 7.2) containing 100 mM of KCl and 1mM EDTA at 298 K.

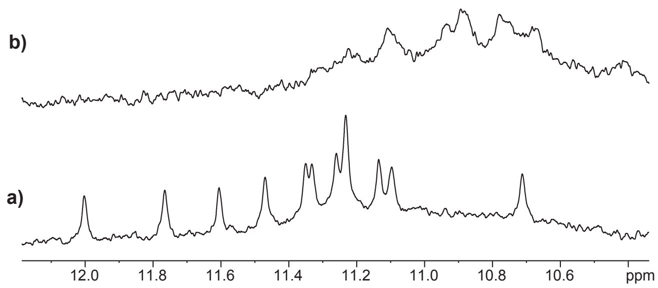

Figure S7: a) The imino region of the ^1H NMR spectrum of the c-kit1 sequence, b) ^1H NMR spectrum recorded after the addition of 1 equivalent of APD displaying a large upfield shift and line broadening. Conditions: 400 μM DNA, 100 mM KCl, 20 mM potassium phosphate buffer (pH 7.4), 298 K, 500 MHz.

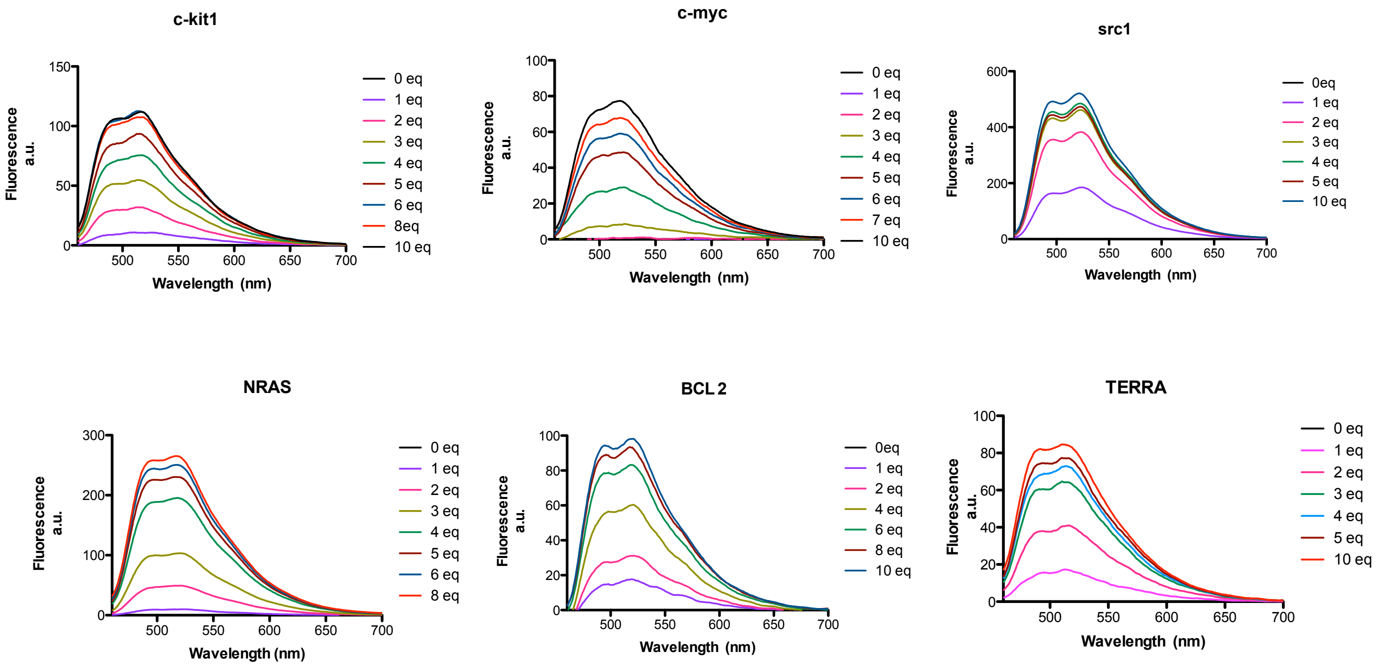

Figure S8: Fluorescence titration of a 0.5 μM solution of APD with increasing amounts of the respective oligonucleotides. Conditions: 0.5 μM APD, 50 mM potassium phosphate buffer (pH 7.4), 50 mM KCl, 298 K.

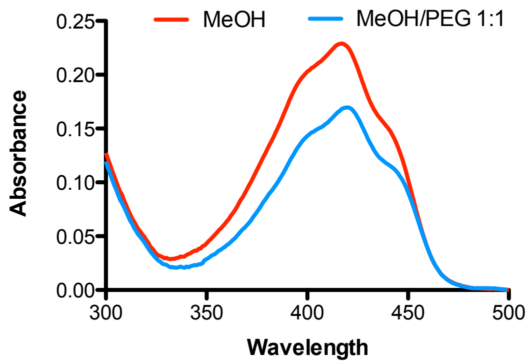

Figure S9: UV-Vis absorption spectra of 25 μM solutions of APD in MeOH and PEG-MeOH (1:1) at 298 K.

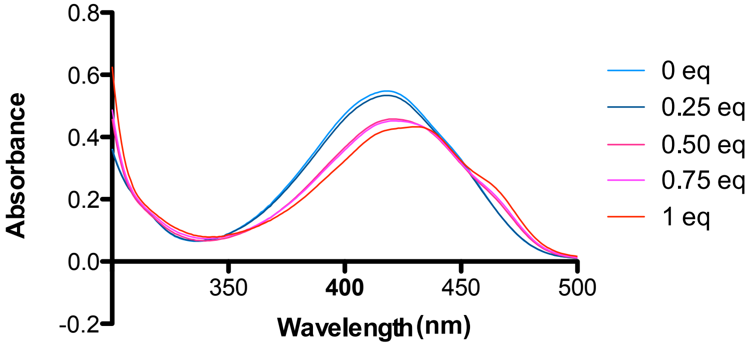

Figure S10: UV titrations of APD with pre-annealed c-kit1. Conditions: 25 μM APD, 50 mM potassium phosphate buffer (pH 7.4), 50 mM KCl, 298 K. The spectral changes stopped after the addition of 1 eq of c-kit1.

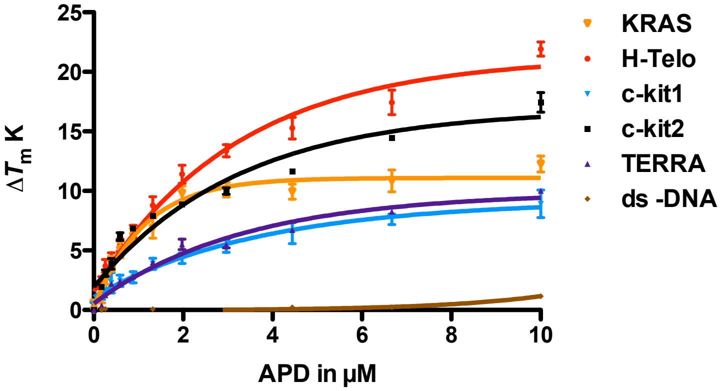

Figure S11: Changes in the melting temperature of a panel of dual labeled oligonucleotides upon addition of increasing concentrations of APD. Conditions: 200 nM oligonucleotides, 60 mM potassium cacodylate buffer, pH 7.4.

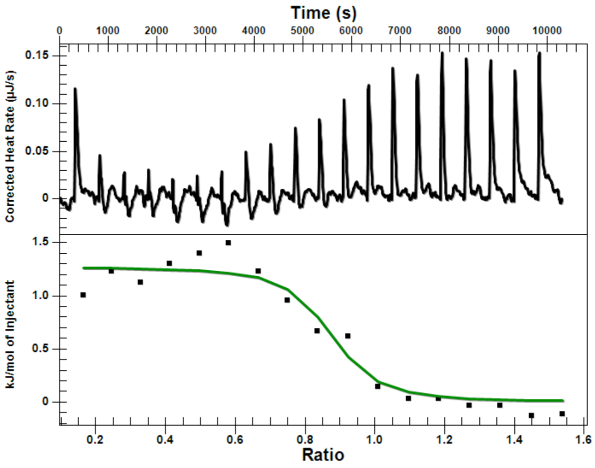

Figure S12: Isothermal calorimetric titrations of c-kit1 with APD. Data were fitted with an independent binding model: dH = (KJ/mol) 1.274, dS = 124.9, K_d = 0.4992 μM, n = 0.936.

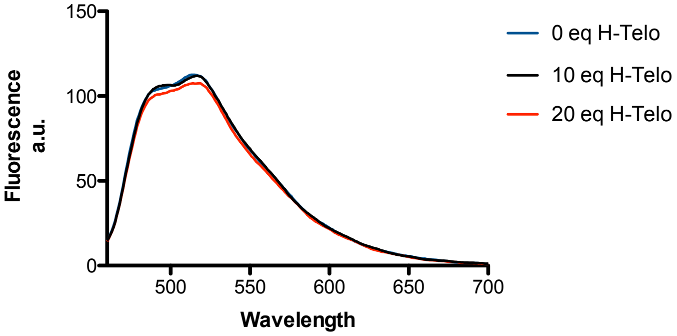

Figure S13: The effect observed in the fluorescence emission a preformed complex of TERRA/APD upon treatment with 20-fold excess of h-Telo. Conditions: 500 nM APD, 500 nM TERRA 50 mM potassium phosphate buffer (pH 7.4), 50 mM KCl, 298 K.

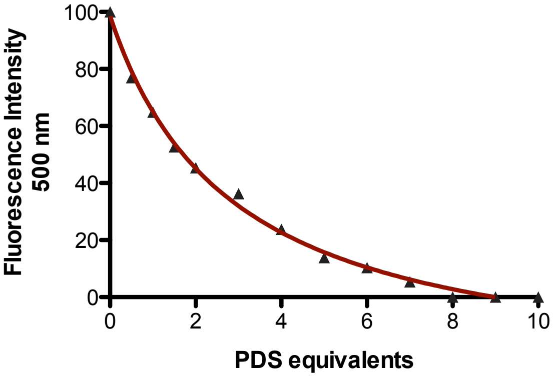

Figure S14: Decrease in the fluorescence emission of APD upon the addition of increasing amount of a competing G-quadruplex ligand (PDS). Conditions: 500 nM APD, 500 nM c-kit1 50 mM potassium phosphate buffer (pH 7.4), 50 mM KCl, 298 K.
